# A novel comprehensive classification system for bicuspid aortic valve morphology at cardiac MRI and initial results in 368 patients

**DOI:** 10.1186/1532-429X-17-S1-P314

**Published:** 2015-02-03

**Authors:** Ian G Murphy, Alex J Barker, Jeremy D Collins, Michael Markl, Colleen Clennon, S C Malaisrie, Patrick M McCarthy, James C Carr

**Affiliations:** 1 Radiology, Northwestern Memorial Healthcare, Chicago, IL, 60613, USA, Chicago, IL, USA

## Background

**Figure 1:**
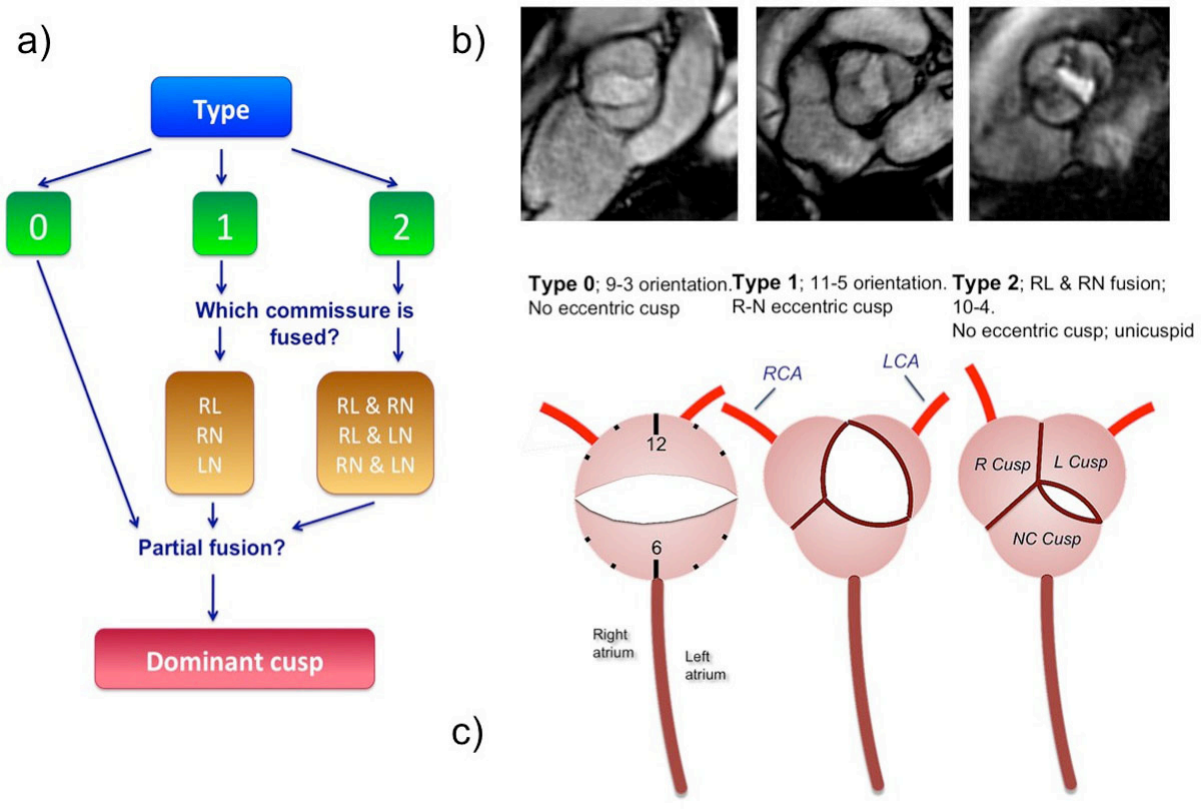


Significant morphological heterogeneity is known to exist among subjects with bicuspid aortic valves (BAV). Accompanying this is the recognition of varied penetrance of phenotypic aortopathy. A genetic contribution possibly influenced by blood flow patterns in the aortic root has been postulated. Current BAV classification schemes do not comprehensively characterize the heterogeneous expression of BAV morphology (Fig 1a). The purpose of this study is to describe a novel comprehensive classification system for BAV morphology in a consecutive cohort of patients referred for evaluation of the thoracic aortic size.

**Figure 2:**
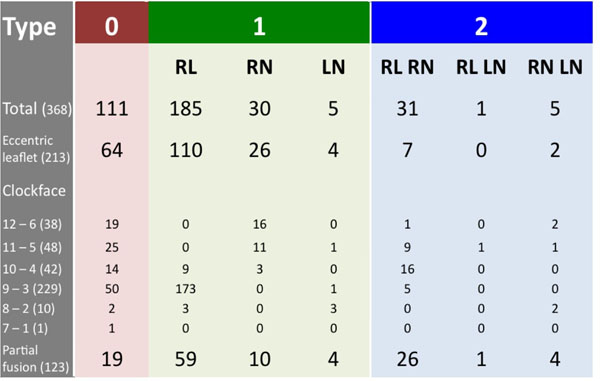


## Methods

Retrospective analysis of MRI data of patients with BAV imaged between November 2012 and August 2014 was carried out. All subjects underwent segmented balanced steady-state free precession (bSSFP) cinegraphic and 2-dimentional through-plane phase-contrast imaging at, below, and above the aortic valve. BAV morphology was categorized by a single observer blinded to the clinical report. The following novel comprehensive classification scheme was applied (Fig 1):

1) Existence of valve raphe as proposed by Sievers et al (2007): type 0 = no visible raphe; type 1 = presence of a raphe, as identified by leaflet ‘fusion', e.g. 1 RL, RN or left-noncoronary (LN); type 2 = presence of 2 raphes.

2) Orientation of the valve orifice opening as using a clock face scheme (Fig 1c), with the inter-atrial septum establishing the 12-6 axis of the clock face.

3) If present, the position of an eccentric / dominant leaflet, during systole (Fig 1b, 1c).

4) If present, the location of partially fused leaflets were noted.

## Results

Of 368 eligible patients, the type 1 RL valve phenotype was the most prevalent morphology with an opening orientation between 8-10 o'clock (table 1, n=185, 50%). When type 0 subjects with a similar opening orientation are included, the 68% incidence of the opening orientation aligns with previous surgical reports (73%, n=304, Sievers et al, J Thorac Cardiovasc Surg. 2007). When combined, the 24% incidence of Type 1 RN and type 0 between 5-7 o'clock aligns well with surgical reports (19%). Of note, eccentric valve leaflets were seen in 213 (58%) and partial fusions were found in 123 (33%) of all cases.

## Conclusions

The presented novel BAV classification scheme enabled comprehensive characterization of valve morphology and orifice orientation while noting the presence of partial leaflet fusion and leaflet asymmetry. Our results demonstrate a comprehensive, non-invasive MRI-based method to categorize BAV morphology, highlighting the considerable heterogeneity of valve morphology. We found that by coarsely grouping leaflet morphology, our leaflet fusion results aligned well with frequency of fusion patterns reported previous studies. Comprehensive characterization of BAV morphology is the first step to understand the influence of valve opening on aortic root hemodynamics, independent of valvular function.

## Funding

No outside funding was required for this study.

